# Disease-gene prediction based on preserving structure network embedding

**DOI:** 10.3389/fnagi.2023.1061892

**Published:** 2023-02-21

**Authors:** Jinlong Ma, Tian Qin, Ju Xiang

**Affiliations:** ^1^School of Information Science and Engineering, Hebei University of Science and Technology, Shijiazhuang, China; ^2^School of Computer and Communication Engineering, Changsha University of Science and Technology, Changsha, China; ^3^Department of Basic Medical Sciences, Changsha Medical University, Changsha, China

**Keywords:** disease-gene prediction, heterogeneous network, network embedding, network propagation, human essential genes

## Abstract

Many diseases, such as Alzheimer's disease (AD) and Parkinson's disease (PD), are caused by abnormalities or mutations of related genes. Many computational methods based on the network relationship between diseases and genes have been proposed to predict potential pathogenic genes. However, how to effectively mine the disease-gene relationship network to predict disease genes better is still an open problem. In this paper, a disease-gene-prediction method based on preserving structure network embedding (PSNE) is introduced. In order to predict pathogenic genes more effectively, a heterogeneous network with multiple types of bio-entities was constructed by integrating disease-gene associations, human protein network, and disease-disease associations. Furthermore, the low-dimension features of nodes extracted from the network were used to reconstruct a new disease-gene heterogeneous network. Compared with other advanced methods, the performance of PSNE has been confirmed more effective in disease-gene prediction. Finally, we applied the PSNE method to predict potential pathogenic genes for age-associated diseases such as AD and PD. We verified the effectiveness of these predicted potential genes by literature verification. Overall, this work provides an effective method for disease-gene prediction, and a series of high-confidence potential pathogenic genes of AD and PD which may be helpful for the experimental discovery of disease genes.

## 1. Introduction

Alzheimer's disease (AD) and Parkinson's disease (PD) are two of the most common forms of neurodegenerative illness that affect people all over the globe. The two diseases are both complicated problems that are associated with aging. AD is the most common cause of dementia as well as other neurodegenerative illnesses, and the symptoms of the condition often include behavioral abnormalities, memory loss, and cognitive impairment (Joe and Ringman, 2019; Li et al., 2021). About 1–2 percent of adults over the age of 60 are diagnosed with PD, making PD the most prevalent motor neurodegenerative illness (Wirdefeldt et al., 2011; Ascherio and Schwarzschild, 2016). Diagnosis of PD can be established when typical features of dyskinesia are combined with other features such as rigidity, tremor, and postural instability (Postuma et al., 2015). For patients, these disease may bring them tremendous emotional pressure and financial pressure. At the moment, patients are mostly treated with pharmaceuticals such as N-methyl-D-aspartic acid receptor antagonists, memantine, and cholinesterase inhibitors. For the time being, there is no all-encompassing therapeutic solution accessible for this condition (Jevtic et al., 2017). Therefore, it is an urgent need to explore and evaluate novel cell-related biomarkers and therapeutic targets to increase the level of diagnosis and therapy offered to patients with AD or PD. The creation of gene networks may bring about the alterations associated with the etiology and development of AD and PD.

The screening and identification of pathogenic genes is one of the research hot spots in the field of modern bio-medicine, which is important to explore the pathogenic mechanism of the disease. The rapid advancement of contemporary biomedical technology has led to the production of a significant amount of data about biological networks, which in turn has facilitated the development of network bio-medicine (Ata et al., 2021; Xiang et al., 2022b). This change has resulted in the development of a novel concept and path for the screening of pathogenic genes. Many network-based algorithms have been proposed to prioritize screening disease-related candidate genes. Hu et al. (2018) proposed a novel disease-gene-prediction method by combining path-based structure with community structure characteristics in human protein–protein networks. Liu et al. (2021) built a biological heterogeneous network on known correlations between the entities from different sets, and proposed a new network embedded representation algorithm to calculate the correlation between disease and genes, using the correlation score to predict pathogenic genes. Xiang et al. (2021a) proposed a framework of network impulsive dynamics on multiplex biological network to predict disease-related genes which could identify disease-related genes by mining the dynamical responses of nodes to impulsive signals being exerted at specific nodes. Xiang et al. (2022a) proposed a hybrid disease-gene prediction method integrating multiscale module structure (HyMM), which could utilize multiscale information from local to global structure to more effectively predict disease-related genes. Ruan and Wang (2021) proposed a Disease-Specific Network Enhancement Prioritization (DiSNEP) framework to improve disease gene prioritization using networks. However, how to effectively mine the disease-gene relationship network to predict disease genes better is still an open problem.

Network embedding, which is an effective way to extract useful information from networks, transforms the nodes of network into low-dimensional spatial vectors while maximizes the information about the network structure and attributes (Mikolov et al., 2013; Perozzi et al., 2014; Tang et al., 2015; Wang et al., 2019). For instance, Li et al. suggested a representation learning method which used joint binary network embedding to conduct an analysis of single-cell RNA-seq data. The proposed heterogeneous network was able to incorporate numerous binary networks, allowing for a low-dimensional representation of a variety of node types to obtain (Li and Patra, 2010). DeepWalk (Perozzi et al., 2014) implements a depth-first search over the network, whereas LINE (Tang et al., 2015) implements a breadth-first searching strategy to generate a context for nodes. Zeng et al. presented the idea of embedding multiview knowledge in order to get an understanding of entity embedding. This was due to the fact that multiview learning might lead to improved generalization performance in order to learn exhaustive entity embedding from various views (Zeng et al., 2016). Xiang et al. (2021b) proposed a method for predicting disease-related genes by using fast network embedding (PrGeFNE), which could integrate multiple types of associations related to diseases and genes.

In this paper, a method called preserved structure network embedding (PSNE) is offered for the prediction of disease genes. Firstly, we collect disease-gene associations, human protein network, and disease-disease associations to construct a heterogeneous network for integrating information. Each node in the heterogeneous network represents a vector that can retain the input network structure. Secondly, the network embedding algorithm is introduced to obtain low-dimensional vector representations of the nodes that make up the network. Thirdly, in order to remove unnecessary information, the low-dimensional vector representations that are retrieved from the nodes are made to be sparse. In order to create the disease-gene network, as well as to rebuild the heterogeneous network made up of diseases and genes, low-dimensional vector representations are used. In the last step, network propagation algorithm is used in order to forecast disease genes utilizing the newly developed two-layer heterogeneous network.

The remaining parts of the article are detailed down below. In Section 2, the disease-related gene data sources used in this investigation are outlined. Then, a method called PSNE is proposed for predicting disease genes. In Section 3, we compare other methods to confirm the excellent performance of PSNE through experiments and analyze the gene prediction ability of the PSNE method in AD and PD. In Section 4, we summarize the work and discussion.

## 2. Materials and methods

### 2.1. DATASET

#### 2.1.1. Disease-gene association

Data availability, fragmentation, heterogeneity and inconsistency of concept description are problems that must be overcome in disease mechanism research. DisGeNet is a database which collects a large number of mutations and genes related to human diseases (Mendelian genetic diseases, complex diseases, and environmental diseases). DisGeNet is a collection of data obtained by collating and combining the data from public databases, scientific literature, Genome-wide association study catalogs, and animal models. The data collected by the database are annotated by a unified standard. In addition, there are more perfect basic criteria to determine the order of the relationship between genotypes and phenotypes. This information can be accessed through web interfaces, cytoscape applications, R packages, and scripting in several programming languages. DisGeNet is not only a multi-functional platform but also can be used for different research purposes, comprising the molecular essential of specific human diseases and their complications, analyzing pathogenic gene characteristics, constructing drug therapeutic effects and hypotheses of adverse drug reactions, testing candidate disease genes, and evaluating text mining approaches. The latest version of DisGeNet is v7.0, containing 1,134,942 genetic disease associations, between 21,671 genes and 30,170 diseases, symptoms, characteristics, and clinical or abnormal human phenotypes, as well as 369,554 variant disease associations, between 194,515 variants and 14,155 diseases, characteristics, and phenotypes.

In this paper, the DisGeNet database is combed in search of disease-gene connections and filtered the primary dataset. Firstly, we choose “disease” as “diseaseType” and “Disease or Syndrome” as “diseaseSemanticType.” Then, the genes that do not exist in the human protein network are filtered out. If a disease has the same set of disease genes, only one of the diseases is randomly retained. Finally, the number of disease-gene associations is 20,274.

#### 2.1.2. Human protein network

To avoid the incompleteness of human protein networks caused by a single data source, we employ extensive interactions that are generated from the sources listed below (Menche et al., 2015). (1) Regulatory interactions; (2) literature-compiled interactions, which are mostly derived from low-throughput trials; (3) binary interactions derived from numerous yeast two-hybrid high-throughput datasets as well as data gathered from the published literature; (4, 5) interactions of the signaling pathways; (6) pair of kinase and substrate molecules; and (7) protein complexes.

#### 2.1.3. Disease-disease association

By using the same method in MimMiner recently, the disease-disease similarity scores are calculated to construct the disease-disease network. The OMIM IDs are mapped one by one to UMLS IDs in DisGeNet, and then k-nearest neighbor method is used to obtain sparse disease-disease network.

### 2.2. Methods

**Figure 1:**
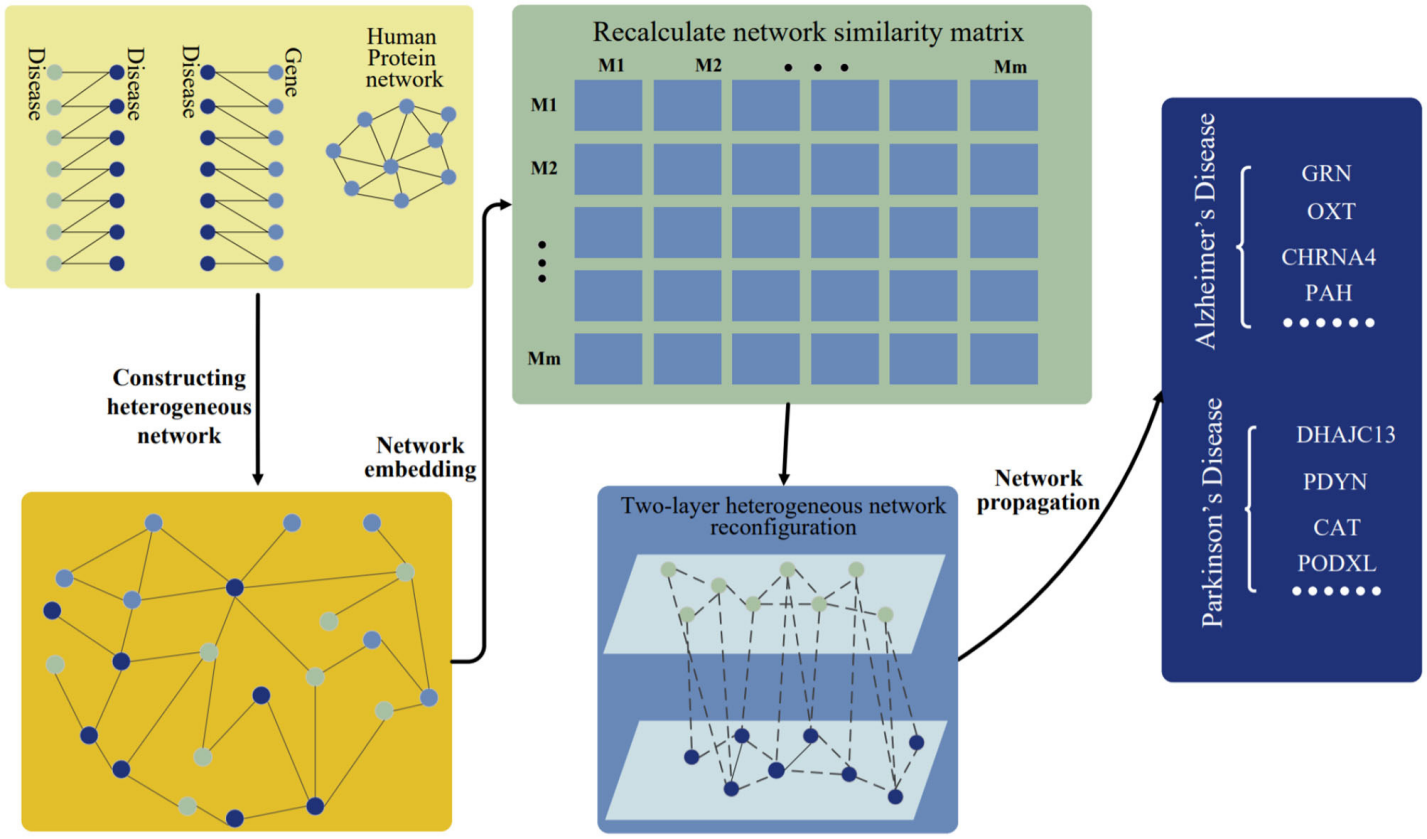
Workflow of PSNE. The disease-disease associations, disease-gene associations, and human protein network are connected together and constructed as a heterogeneous network. The network embedding algorithm is used to realize the low-dimensional vector representation of the network and then a new two-layer heterogeneous network of diseases and genes is constructed. Finally, the network propagation algorithm is used to predict the pathogenic genes.

Here, the disease-gene prediction method is presented by using preserving structure network embedding (PSNE), which can use multi-source biological information to predict disease-related genes more effectively. The PSNE method consists of four parts: heterogeneous network construction, network embedding algorithm, heterogeneous network reconfiguration and heterogeneous network propagation (see Figure 1). Next, we will describe the details of the four parts.

#### 2.2.1. Heterogeneous network construction

The disease gene prediction method is get start by constructing heterogeneous networks using disease and gene association data from multiple sources. In order to solve the network sparsity problem, the disease-gene network is needed to enrich by using other known human protein relationship networks and disease-disease relationships. Heterogeneous network is constructed by integrating three different types of connected data: disease-gene associations, human protein network, and disease-disease associations.

#### 2.2.2. Network embedding algorithm

Network embedding is an algorithm to get the information from the network, which converts the nodes into a vector of low dimensional space while maximally preserves the network structural information (Dai et al., 2019). There are many network embedding algorithms. The random walk algorithm is used in the PSNE method. The purpose of network embedding is to determine the interconnections that exist between each node and the links that are immediately around the node by using a series of vectors that is created by random walk (Grover and Leskovec, 2016). The functional similarity of two nodes in a network is correlated with the distance between those nodes. Network analysis is utilized to provide a quantitative assessment of the links between genes and diseases. More researches can be done in the disease-gene associations by applying the network embedding algorithm to the structure.

Let *G* = (*V, E, W*) denote a heterogeneous network, where vertex *v* ∈ *V* indicates a gene, *e*(*u, v*) ∈ *E* is the edge of connecting genes *v* and *u*, *w*(*u, v*) ∈ *W* is the weight of edge *e*(*u, v*), which is used to characterize the probability of a relationship between *u* and *v*. Here, the weight of all edges in the heterogeneous network is set to 1, which means that they are equal to each other. Considering the nature of grouping between heterogeneous networks, the vertex may move toward its adjacent position with different probabilities. In the process of random walk, nodes tend to travel along the edge and have the highest probability of transitioning to their proximity. Given a vertex *v*_*i*_ and an edge *e*(*v*_*i*−1_, *v*_*i*_), where vertex *v*_*i*_ visited vertex *v*_*i*−1_ in the previous step. By calculating the transition probability *T*(*v*_*i*_, *v*_*i*+1_) on edge *e*(*v*_*i*_, *v*_*i*+1_), vertex *v* is transferred to one of its neighbors *v*_*i*+1_. The transition probability *T*(*v*_*i*_, *v*_*i*+1_) is defined as follows:


(1) $$\begin{array}{l} T\left(v_{i},v_{i+1}\right)=\pi \left(v_{i-1},v_{i+1}\right)*W, \end{array}$$

(2) $$\pi (v_{i-1},v_{i+1})\left\{ \begin{array}{l} \frac{1}{p},d_{v_{i-1}v_{i+1}}=0\\ 1,d_{v_{i-1}v_{i+1}}=1\\ \frac{1}{q},d_{v_{i-1}v_{i+1}}=2, \end{array}\right.$$

where *d*_*v*_*i*−1_*v*_*i*+1__ represents the shortest path distance from the previous vertex *v*_*i*−1_ to the next vertex *v*_*i*+1_. *d*_*v*_*i*−1_*v*_*i*+1__ = 0 means that vertex *v*_*i*−1_ and *v*_*i*+1_ are the same vertex, and vertex *v* jumps back to its previous vertex *v*_*i*−1_, *d*_*v*_*i*−1_*v*_*i*+1__ = 1 means that vertex *v*_*i*+1_ is the common neighbor of vertex *v*_*i*−1_ and *v*, *d*_*v*_*i*−1_*v*_*i*+1__ = 2 means that *v*_*i*−1_ and *v*_*i*+1_ are indirectly connected, and vertex *v*_*i*+1_ is not their common neighbor. The parameter *p* controls the possibility of revisiting the node during random walk. Setting the parameter *p* to a higher value can ensure that we avoid sampling the nodes that have been visited in the next step. This setting encourages moderate exploration and avoids the redundancy of sampling. If the value of parameter *p* is very low, it will cause the walk to backtrack one step, which will make the walk close to the starting node. If the parameter *q* is greater than 1, random walk will tend to be between nodes around the starting point. Such random walk behavior reflects breadth first search. On the contrary, if the parameter *q* is less than 1, random walk is more likely to visit nodes far away from the node, which reflects depth first search. After calculating the transition probability of each edge in the heterogeneous network, the normalized transition probability matrix *T*_*norm*_ is defined to ensure that the sum of the exit probability of each node is 1,


(3) $$\begin{array}{l} T_{norm}\left(v_{i},v_{i+1}\right)=\frac{T\left(v_{i},v_{i+1}\right)}{\sum_{j\in i}T\left(v_{i},v_{j}\right)}. \end{array}$$

Let *G* = (*V, E, W*) denote a heterogeneous network. Let *f* denote the mapping function from node to feature representation to learn the downstream prediction task. Here, *d* is a parameter that specifies the dimension of feature representation, which is set to 128. Equivalently, *f* is a parameter matrix of size |*V*| × *d*. For each source node *u* ∈ *V*, *N*_*S*_(*u*)⊂*V* is defined as the network neighborhood generated by node *u* through domain sampling strategy *S*. Feature learning in networks is described as a model that maximizes the log-probability(log*Pr*) of neighbors *N*_*S*_(*u*),


(4) $$\begin{array}{l} \underset{f}{\max}\underset{u\in V}{\sum }\log Pr\left(N_{S}\left(u\right)|f\left(u\right)\right). \end{array}$$

Two standard assumptions are made in order to help the optimization problem easy to handle. One is conditional independence. Given the characteristic representation of nodes, the possibility is decomposed by assuming the possibility of observing neighborhood nodes is independent of observing any other neighborhood nodes:


(5) $$\begin{array}{l} Pr\left(N_{S}\left(u\right)|f\left(u\right)\right)=\underset{n_{i}\in N_{S}\left(u\right)}{\prod }Pr\left(n_{i}|f\left(u\right)\right), \end{array}$$

And the other is symmetry of feature space. Source nodes and neighborhood nodes have symmetrical influence on each other in the feature space. Therefore, the condition of each node pair is modeled as a unit, and parameterize the node pair through the point product of their characteristics:


(6) $$\begin{array}{l} Pr\left(n_{i}|f\left(u\right)\right)=\frac{\exp\left(f\left(n_{i}\right)\cdot f\left(u\right)\right)}{\sum_{v\in V}\exp\left(f\left(v\right)\cdot f\left(u\right)\right)}. \end{array}$$

With the above assumptions, the objective in Equation 4 is simplified as:


(7) $$\begin{array}{l} \underset{f}{\max}\underset{u\in V}{\sum }\left[-\log Z_{u}+\underset{n_{i}\in N_{S}\left(u\right)}{\sum }f\left(n_{i}\right)\cdot f\left(u\right)\right]. \end{array}$$

For large scale networks, the calculation cost of partition function $Z_{u}=\underset{v\in V}{\sum }\exp\left(f\left(u\right)\cdot f\left(v\right)\right)$ of each node is very high, the negative sampling is used to approximate the partition function *Z*_*u*_. The stochastic gradient ascent algorithm is used to optimize the Equation 7 on the model parameters that define the feature *f*.

#### 2.2.3. Heterogeneous network reconfiguration

Network structures are concise and efficient data structures, which are used to describe related problems in bio-informatics (Hohmann, 2010). The connections between nodes represent their interactions, such as diseases caused by gene expression, reactions between proteins and other interactions. If two nodes in the network are connected, the information shown by the two nodes is considered related. The heterogeneous network representation learning algorithm embeds the rich structural and semantic information in the heterogeneous network into the low-dimensional node representation, which is convenient for downstream applications. Low-dimensional node vectors represent useful high-level correlation information in heterogeneous networks. The data are used to reconstruct a two-layer heterogeneous network. In specifically, $C_{i,j}=\frac{{\vec{s}}_{i}^{T}\cdot {\vec{s}}_{j}}{\left|{\vec{s}}_{i}|\cdot |{\vec{s}}_{j}\right|}$ is used to calculate the cosine similarity between different diseases, where $\vec{s}$ represents the embedding vector of node and ${\vec{s}}^{T}$ is the transposition of $\vec{s}$, and the network embedding algorithm is used to the enhanced disease network. Similarly, an enhanced gene network is made. After that, there is the revised gene association network as well as the disease association network, and disease-gene network is integrated to form a new disease-gene two-layer heterogeneous network *D*, which can be represented by,


(8) $$\begin{array}{l} D=\left(\begin{array}{cc} H_{I} & H_{G}\\ H_{G}^{T} & H_{N} \end{array}\right), \end{array}$$

where *H*_*I*_ represents the improved disease network, *H*_*N*_ represents an improved gene network, *H*_*G*_ indicates a disease-gene association and $H_{G}^{T}$ is the transposition of *H*_*G*_. In contrast to the heterogeneous networks described earlier, the two-layer heterogeneous networks include more concentrated and more pertinent information.

#### 2.2.4. Heterogeneous network propagation

The network propagation is simulated in the new two-layer heterogeneous network of diseases and genes to determine the likelihood of disease-associated genes. A disease network *H*_*I*_, a gene network *H*_*N*_, a disease-gene network *H*_*G*_ and *H*_*E*_ which is the transposition of *H*_*G*_ are given, each of them is denoted by a symbol. Then, we come up with the following diagonal matrix *D*_*I*_, *D*_*N*_, *D*_*G*_ and *D*_*E*_, where the elements of the diagonal are specified by ${\left(D_{I}\right)}_{i,j}=\underset{j}{\sum }{\left(H_{I}\right)}_{j,i}$, ${\left(D_{N}\right)}_{i,j}=\underset{j}{\sum }{\left(H_{N}\right)}_{j,i}$, ${\left(D_{G}\right)}_{i,j}=\underset{j}{\sum }{\left(H_{G}\right)}_{j,i}$ and ${\left(D_{E}\right)}_{i,j}=\underset{j}{\sum }{\left(H_{E}\right)}_{j,i}$. Then, the normalized matrices of *H*_*I*_, *H*_*N*_, *H*_*G*_ and *H*_*E*_ can be written as,


(9) $$\begin{array}{l} {\hat{H}}_{I}=H_{I}D_{I}^{-1}\\ {\hat{H}}_{N}=H_{N}D_{N}^{-1}\\ {\hat{H}}_{G}=H_{G}D_{G}^{-1}\\ {\hat{H}}_{E}=H_{E}D_{E}^{-1} \end{array}\}$$

These normalized matrices allow us to construct a new matrix.


(10) $$\begin{array}{l} \hat{D}=\left(\begin{array}{cc} (1-\beta ){\hat{H}}_{I} & \beta {\hat{H}}_{G}\\ \beta {\hat{H}}_{E} & (1-\beta ){\hat{H}}_{N} \end{array}\right), \end{array}$$

where β is the jump probability between layers. The random walk can jump to the gene network with probability β when applying to the disease network, or remain in the original network with the probability 1−β.

The inter-layer jump can take place only when the node reached by the random walk algorithm is linked to the node of another layer's. In such case, node can only be moved to that is close to the node in the layer, or may be taken back to the node where the node began. In light of this, we refer to a diagonal matrix as *H*_Ḋ_ and ${\left(H_{Ḋ}\right)}_{i,i}=\underset{j}{\sum }{\left(\hat{D}\right)}_{j,j}$. In a two-layer heterogeneous network, the following approaches is used to get the final transfer matrix *T* of the network propagation process,


(11) $$\begin{array}{l} T=\hat{D}\cdot H_{\hat{D}}^{-1}. \end{array}$$

The model of a random walk with a reset can be described by,


(12) $$\begin{array}{l} q_{t+1}=\left(1-\alpha \right)T\cdot q_{t}+\alpha q_{0}. \end{array}$$

The initial probability vector of the random walk is characterized by $q_{0}={\left(q_{0D}^{T},q_{0G}^{T}\right)}^{T}$, where $q_{0D}^{T}$ is the initial probability vector of the disease network. The initial probability vector of the gene network is denoted by $q_{0G}^{T}$, where parameter α ∈ (0, 1) is the restart probability, which means that the random walk algorithm will have probability α to return to the initial position. After a certain number of steps, the acquired probability will eventually arrive at a state of stability. The genes can be sequenced in accordance with the probability which will stabilize, and then the genes can be predicted which are associated with the diseases.

## 3. Results

### 3.1. Evaluation parameters

In this section, we utilize the disease gene association network that is included inside DisGeNet as a standard dataset to assess the effectiveness of PSNE. In addition, a number of traditional well-performance disease gene prediction methods, such as dgn2vec, PRINCE (Vanunu et al., 2010), CIPHER (Wu et al., 2008), BiRW (Xie et al., 2015), RWR (Köhler et al., 2008) and DK (Köhler et al., 2008) are used as comparison.

In the comparison of performance, all other genes are used as control group except for genes related to diseases in training and testing groups, which are called genome-wide control set. AUROC, AUPRC and top-K Recall and Precision then are used as evaluation parameters (Vihinen, 2012). AUROC is defined as the area under the receiver's operating characteristic curve and AUPRC is defined as the area under the precision-recall curve, the two parameters are able to use the intuitive indications of the data in order to assess the performance of the classifiers, and effective reflection of predictive sequencing of disease-associated genes is possible. The greater score means that the performance of the classifier is better. For the disease *d* in the disease set *D*, *T*_*D*_ represents the set of genes in the test set. The disease gene prediction algorithm will provide a ranking list of candidate genes for disease *d*. We use *R*_*D*_(*k*) to represent the collection of the first *k* candidate genes in the ranking list. Then, Recall in the top-k ranking list is set as Recall = |*T*_*d*_ ∩ *R*_*d*_(*k*)| / |*T*_*d*_|, and Precision in the top-k ranking list is set as Precision = |*T*_*d*_ ∩ *R*_*d*_(*k*)| / |*R*_*d*_(*k*)|.

### 3.2. Overall comparison

**Figure 2:**
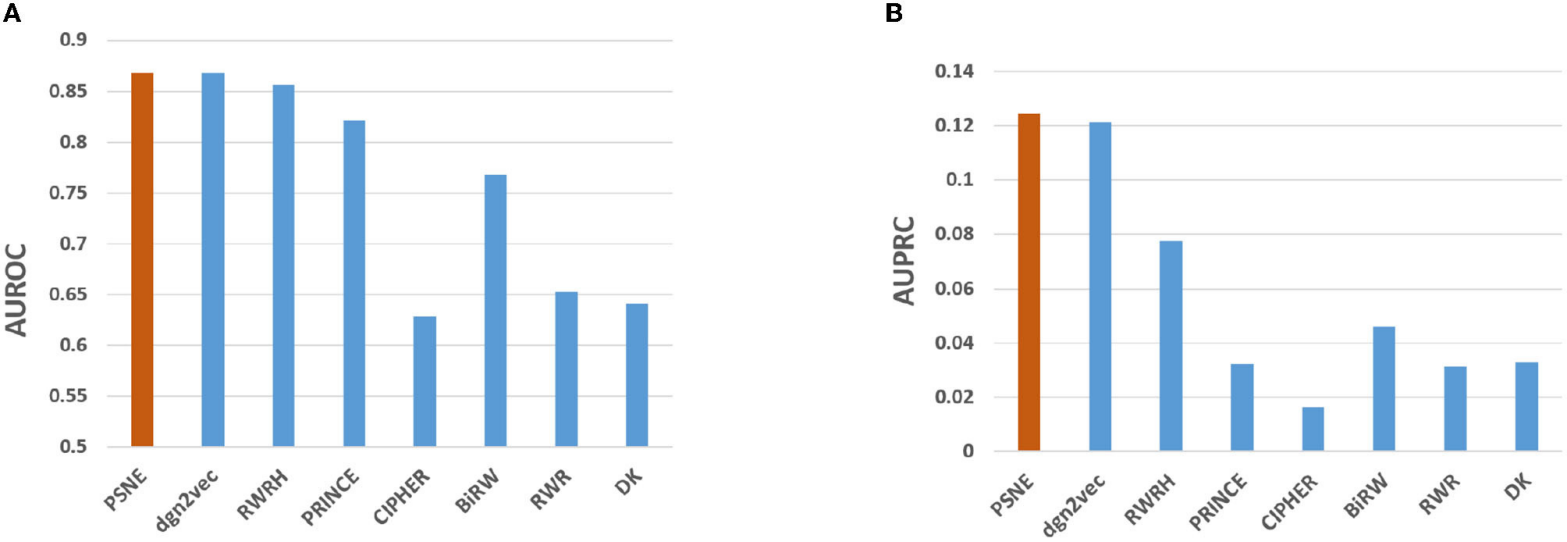
Performance evaluation of PSNE in terms of **(A)** AUROC and **(B)** AUPRC, with comparison to the state-of-the-art methods.

Figure 2 shows the AUROC and AUPRC values of PSNE, dgn2vec, RWRH, PRINCE, CIPHER, BiRW, RWR and DK where AUROC values are 0.868, 0.867, 0.856, 0.821, 0.628, 0.768, 0.653, 0.641; AUPRC values are 0.125, 0.121, 0.078, 0.032, 0.016, 0.046, 0.031, and 0.032, respectively. Compared with other advanced methods, PSNE has different degrees of improvement, with a maximum increase of 27.5% (AUROC) and 74.8% (AUPRC). Table 1 shows that the top-k Recall rates for PSNE are 0.078, 0.176, 0.227, 0.287, 0.359, 0.418, and 0.499. In the top-k Recall rate, PSNE is better than all comparison methods.

**Table 1:** Performance evaluation of PSNE in terms of top-k Recall (k = 1, 5, 10, 50, 100, and 200), with comparison to the state-of-the-art methods.

**Methods**	**Recall_1**	**Recall_5**	**Recall_10**	**Recall_20**	**Recall_50**	**Recall_100**	**Recall_200**
PSNE	**0.078**	**0.176**	**0.227**	**0.287**	**0.359**	**0.418**	**0.499**
dgn2vec	0.075	0.174	0.223	0.279	0.349	0.411	0.497
RWRH	0.046	0.121	0.172	0.237	0.322	0.393	0.475
PRINCE	0.015	0.057	0.093	0.143	0.211	0.257	0.307
CIPHER	0.007	0.029	0.049	0.081	0.137	0.184	0.232
BiRW	0.027	0.080	0.117	0.164	0.234	0.286	0.432
RWR	0.019	0.056	0.077	0.102	0.137	0.166	0.195
DK	0.021	0.058	0.077	0.101	0.131	0.155	0.182

Bold values represent the maximum values in each column of data.

### 3.3. Comparison of the results and case study for Alzheimer's disease

Tables 2, 3 show that the top-k Recall and Precision rates of PSNE and other methods. It is also obvious from the figure that the performance of PSNE is superior to other methods. To further illustrate the performance of PSNE, we used PSNE method to predict and analyze the genes that may cause AD. Table 4 shows the top 20 Alzheimer's-related genes predicted by PSNE and the corresponding literature support. Progranin encoded by GRN gene, plays a key role in the development, survival, function and maintenance of neurons and microglia in mammalian brain. GRN functional deletion mutations cause neuronal waxy lipofuscinosis or frontotemporal dementia-GRN (FTD-GRN) in a dose-dependent manner. Mutations that lower PGRN levels increase the risk of AD (Rhinn et al., 2022). The implication of genome-wide significant differential methylation of OXT, encoding oxytocin, in two independent cohorts indicates it is a promising target for future studies on early biomarkers and novel therapeutic strategies in AD (Lardenoije et al., 2019). Ma et al. (2015) suggested that targeting TYROBP might provide a new opportunity for the treatment of AD based on its potential protective role in the pathogenesis of AD. The report from Marioni et al. (2019) evidence that the association of SNPs in the TOMM40 gene with AD is potentially mediated by both gene expression and DNA methylation in the prefrontal cortex.

**Table 2:** For Alzheimer's disease, the performance evaluation of PSNE in terms of top-k Recall (k = 1, 5, 10, 50, 100, and 200) with comparison to the state-of-the-art methods.

**Methods**	**Recall_1**	**Recall_5**	**Recall_10**	**Recall_20**	**Recall_50**	**Recall_100**	**Recall_200**
PSNE	**0.022**	**0.042**	**0.062**	**0.132**	**0.222**	**0.325**	**0.467**
dgn2vec	0.008	0.037	0.053	0.073	0.160	0.245	0.352
RWRH	0.017	0.026	0.042	0.087	0.149	0.269	0.412
PRINCE	0	0.004	0.009	0.009	0.016	0.085	0.164
CIPHER	0	0	0	0	0.006	0.018	0.052
BiRW	0	0	0.007	0.035	0.110	0.197	0.294
RWR	0.008	0.013	0.013	0.029	0.072	0.146	0.265
DK	0.015	0.031	0.057	0.077	0.146	0.215	0.290

Bold values represent the maximum values in each column of data.

**Table 3:** For Alzheimer's disease, the performance evaluation of PSNE in terms of top-k Precision (k = 1, 5, 10, 50, 100, and 200), with comparison to the state-of-the-art methods.

**Methods**	**Prec_1**	**Prec_5**	**Prec_10**	**Prec_20**	**Prec_50**	**Prec_100**	**Prec_200**
PSNE	**0.320**	**0.136**	**0.100**	**0.106**	**0.069**	**0.050**	**0.036**
dgn2vec	0.120	0.104	0.080	0.058	0.050	0.038	0.027
RWRH	0.240	0.080	0.064	0.070	0.047	0.041	0.032
PRINCE	0	0.016	0.016	0.008	0.006	0.013	0.013
CIPHER	0	0	0	0	0.002	0.003	0.004
BiRW	0	0	0.012	0.028	0.035	0.031	0.022
RWR	0.120	0.040	0.020	0.024	0.023	0.023	0.020
DK	0.240	0.104	0.092	0.060	0.046	0.034	0.022

Bold values represent the maximum values in each column of data.

**Table 4:** Top 20 related genes of AD predicted by PSNE.

**Top 1–10 gene**	**Evidence**	**Top 11–20 gene**	**Evidence**
GRN	PMID: 35039149	POMC	PMID: 32982666
OXT	PMID: 31775875	TOMM40	PMID: 29777097
CHRNA4	PMID: 23701948	PON1	PMID: 30714958
PAH	PMID: 27294413	ATP13A2	PMID: 25056458
TYROBP	PMID: 25052481	SOD1	PMID: 33402227
IL6	PMID: 30104698	END1	PMID: 33723589
TFB1M	PMID: 32497722	CAT	PMID: 27809706
NOS2	PMID: 32087283	NGF	PMID: 30804738
IFNG	PMID: 20213229	IL10	PMID: 31879236
CBS	PMID: 32754109	PTGS2	PMID: 29784049

### 3.4. Comparison of the results and case study of Parkinson's disease

Tables 5, 6 show that the top-k Recall and Precision rates of PSNE and other methods. Except that Recall-1 and Precision-1 is not higher than dgn2vec and RWRH, the effect of PSNE is better than other methods. Generally speaking, PSNE also has advantage in the prediction of Parkinson's-related genes.

**Table 5:** For Parkinson's disease, the performance evaluation of PSNE in terms of top-k Recall (k = 1, 5, 10, 50, 100, and 200), with comparison to the state-of-the-art methods.

**Methods**	**Recall_1**	**Recall_5**	**Recall_10**	**Recall_20**	**Recall_50**	**Recall_100**	**Recall_200**
PSNE	0.008	**0.067**	**0.119**	**0.188**	**0.324**	**0.430**	**0.510**
dgn2vec	**0.014**	0.064	0.089	0.148	0.298	0.403	0.489
RWRH	0.012	0.020	0.043	0.069	0.140	0.235	0.381
PRINCE	0	0	0	0.027	0.038	0.056	0.078
CIPHER	0	0	0	0	0	0.006	0.043
BiRW	0	0	0	0.027	0.038	0.056	0.079
RWR	0	0.002	0.010	0.020	0.041	0.051	0.093
DK	0	0.002	0.006	0.013	0.028	0.044	0.056

Bold values represent the maximum values in each column of data.

**Table 6:** For Parkinson's disease, the performance evaluation of PSNE in terms of top-k Precision (k = 1, 5, 10, 50, 100, and 200), with comparison to the state-of-the-art methods.

**Methods**	**Prec_1**	**Prec_5**	**Prec_10**	**Prec_20**	**Prec_50**	**Prec_100**	**Prec_200**
PSNE	0.160	**0.264**	**0.240**	**0.192**	**0.134**	**0.089**	**0.052**
dgn2vec	**0.280**	0.264	0.180	0.150	0.123	0.083	0.050
RWRH	0.240	0.088	0.088	0.069	0.058	0.049	0.039
PRINCE	0	0	0	0.028	0.016	0.012	0.008
CIPHER	0	0	0	0	0	0.001	0.005
BiRW	0	0	0	0.028	0.016	0.012	0.008
RWR	0	0.008	0.020	0.020	0.018	0.011	0.010
DK	0	0.008	0.012	0.014	0.012	0.010	0.006

Bold values represent the maximum values in each column of data.

Likewise, we used the PSNE method to predict and analyze the genes that may cause PD. Table 7 shows the top 20 Parkinson's-related genes predicted by PSNE and the corresponding literature support. In late-onset disease which is most reminiscent of idiopathic PD subtle deficits in endosomal receptor-sorting/recycling are highlighted by the discovery of pathogenic mutations DNAJC13. Molecular deficits in these processes are genetically linked to the phenotypic spectrum of Parkinsonism associated with Lewy body pathology (Vilariño-Güell et al., 2014). Nitric oxide synthase (NOS) genes (NOS1, NOS2A, and NOS3) may create excess nitric oxide that contributes to neurodegeneration in Parkinson's disease (PD). NOS genes might also interact with one another or with environmental factors in PD (Hancock et al., 2008). The protein product of the nuclear-encoded POLG gene plays a key role in the maintenance of mitochondrial DNA replication, and its failure causes multi-system diseases with varying severity. It is known that mitochondrial dysfunction in Parkinson's disease plays a key role in the loss of dopaminergic neurons in the substantia nigra. Therefore, changes in the POLG gene may influence the development of various hereditary neurodegenerative diseases, including monogenic Parkinsonism (Illés et al., 2020).

**Table 7:** Top 20 related genes of PD predicted by PSNE.

**Top 1–10 gene**	**Evidence**	**Top 11–20 gene**	**Evidence**
DNAJC13	PMID: 24218364	NOS3	PMID: 18663495
PDYN	PMID: 17203488	NOS2	PMID: 32003282
CAT	–	KMT2B	–
PODXL	PMID: 26864383	SERPINA1	PMID: 27184740
GSR	PMID: 30156440	C2orf50	–
DRD4	PMID: 23232665	ADH1C	PMID: 15642852
IL1B	PMID: 32003282	GLUD2	PMID: 33093440
NTRK2	PMID: 31991178	XK	–
ALDH5A1	–	POLG	PMID: 32364361
TH	–	PON1	PMID: 32002976

## 4. Conclusion

In the context of the globalization of AD and PD, it is crucial to identify and predict the pathogenic genes of AD and PD for disease prevention and treatment. In this paper, we first combined a disease-gene network, disease-disease network, and human protein network to build a heterogeneous network model, used a network embedding algorithm to achieve low dimensional vector representation of the network. In network embedding algorithm, nodes tended to walk along the edge with the highest transition probability to their neighbors. Assuming that a node had *n* neighbors, it took *O*(*N*) time to find the exit edge with the highest transition probability. Then, a new two-layer heterogeneous network of diseases and genes was constructed. Finally, the network propagation algorithm was used to predict the disease genes. Unlike previous methods of referring to the topological features of heterogeneous protein networks or learning features from gene sequences, this method represented nodes in heterogeneous networks as potential feature vectors. It used network embedding to maximize cross-relationships. We applied the network embedding algorithm to the constructed dataset, and the results showed that our method can achieve better prediction performance. At the same time, we used this method to predict the candidate genes related to AD and PD and carried out literature verification through the PubMed website. We confirmed that most of the predicted candidate genes correlate with AD and PD. In addition, a small number of candidate genes had not been proven on the PubMed website, but at the same time, there was no objection. Perhaps these tiny numbers of genes without examples could provide helpful ideas for the medical research of AD and PD.

## Data availability statement

Publicly available datasets were analyzed in this study. This data can be found here: https://github.com/TianTianTian14/PSNE.

## Author contributions

JM and JX conceived, designed, managed, and reviewed the manuscript. TQ performed the experiments and drafted the manuscript. All authors approved the final manuscript.
